# Comparison between Surgical Access and Percutaneous Closure Device in 787 Patients Undergoing Transcatheter Aortic Valve Replacement

**DOI:** 10.3390/jcm10071344

**Published:** 2021-03-24

**Authors:** Dennis Eckner, Francesco Pollari, Giuseppe Santarpino, Jürgen Jessl, Johannes Schwab, Kristinko Martinovic, Helmut Mair, Matthias Pauschinger, Theodor Fischlein, Ferdinand Vogt

**Affiliations:** 1 Department of Cardiology, Paracelsus Medical University, 90471 Nuremberg, Germany; dennis.eckner@klinikum-nuernberg.de (D.E.); juergen.jessl@klinikum-nuernberg.de (J.J.); johannes.schwab@klinikum-nuernberg.de (J.S.); kristinko.martinovic@klinikum-nuernberg.de (K.M.); matthias.pauschinger@klinikum-nuernberg.de (M.P.); 2 Department of Cardiac Surgery, Paracelsus Medical University, 90471 Nuremberg, Germany; francesco.pollari@klinikum-nuernberg.de (F.P.); theodor.fischlein@klinikum-nuernberg.de (T.F.); 3 Anthea Hospital, GVM Care & Research, 70124 Bari, Italy; gsantarpino@gvmnet.it or; 4 Cardiac Surgery Unit, Department of Experimental and Clinical Medicine, “Magna Graecia” University of Catanzaro, 88100 Catanzaro, Italy; 5 Paracelsus Medical University, 90419 Nuremberg, Germany; 6 Artemed Clinic Munich South, 81379 Munich, Germany; helmut.mair@artemed.de

**Keywords:** transcatheter aortic valve replacement (TAVR), vascular complications, Peripheral Artery Disease (PAD), Aortic Valve Disease, heart team

## Abstract

Background: The vascular access in transcatheter aortic valve replacement (TAVR) was initially dominated by a surgical approach. Meanwhile, percutaneous closure systems became a well-established alternative. The aim of this study was to compare the clinical outcome between the two approaches. Methods: In this retrospective study, we observed 787 patients undergoing a TAVR-Procedure between 2013 and 2019. Of those, 338 patients were treated with surgical access and 449 with the Perclose ProGlide™-System (Abbott, Chicago, IL, USA). According to the Bleeding Academic Research Consortium (BARC) and Valve Academic Research Consortium (VARC) criteria, the primary combined endpoints were defined. Results: Overall hospital mortality was 2.8% with no significant difference between surgical (3.8%) and percutaneous (2.2%) access (*p* = 0.182). Major vascular complications or bleeding defined as the primary combined endpoint was not significantly different in either group (Surgical group 5.3%, ProGlide group 5.1%, *p* = 0.899). In the ProGlide group, women with pre-existing peripheral artery disease (PAD) were significantly more often affected by a vascular complication (*p* = 0.001 for female sex and *p* = 0.03 for PAD). Conclusions: We were able to show that the use of both accesses is safe. However, the surgical access route should also be considered in case of peripheral artery disease.

## 1. Introduction

The number of transcatheter aortic valve replacement (TAVR) implantations has increased steadily in years while conventional aortic valve replacement remained constant. After implementing TAVR procedures in high-risk patients, the range of indications has been extended to intermediate and low-risk patients [1,2,3,4]. In this expansion of the indication, it seems very important to detect and minimize the procedure’s possible risk factors. Advanced valve systems reduced the rate of paravalvular leakage and conduction disorders, cerebral protection systems were used to reduce periinterventional strokes, while smaller sheath designs targeted a reduction in vascular damage.

To reduce the vascular access injury, another critical factor is the vascular access path, which, according to the literature, continues to be one potential cause for a considerable complication rate and mortality [5]. The Bleeding Academic Research Consortium (BARC) and Valve Academic Research Consortium (VARC-2) criteria were defined and used in this study [6]. Major vascular complication results in a long-term increase in mortality. Thus, solutions have to be found to reduce these, especially in younger and low-risk patients [7].

Initially, the surgical approach to the femoral artery was the standard approach, while percutaneous closure devices have been established increasingly. In our study, both groups have been compared to find possible predictors for relevant vascular damage. The results should help to identify patients who benefit most from either option.

## 2. Materials and Methods

Between 2013 to 2019, a total of 787 patients were identified who underwent surgical vascular access or a percutaneous procedure with the Perclose ProGlide™ system (Abbott, Chicago, IL, USA) when undergoing TAVR.

**Figure 1:**
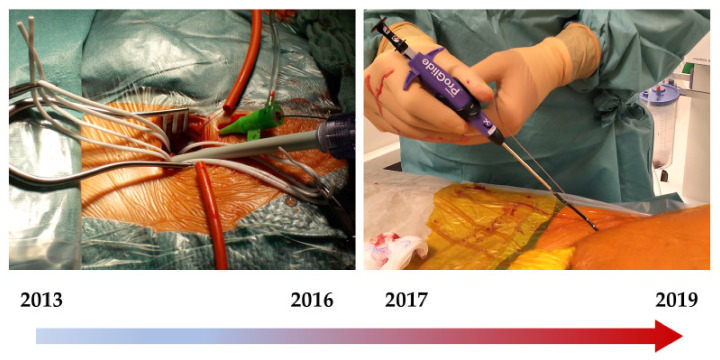
Surgical und ProGlide access over time (intraprocedural view).

Importantly, from 2013 till 2016, all transfemoral interventions were performed using a surgical approach at the Klinikum Nürnberg / Cardiovascular-Center, Nuremberg, Germany, while percutaneous systems were implemented starting in 2017 (Figure 1).

Patients referred to our Center with severe aortic valve stenosis underwent cardiological pre-diagnosis, including echocardiography and invasive coronary diagnostic. The primary decision on femoral access was made by a “TAVR-CT-Scan” that each patient received (Siemens, Berlin, Germany). In addition to the vascular calcification and distribution of the calcification, the vascular diameter was also assessed. A minimum diameter of the femoral artery of 5.5 mm was not undercut. Relevant stenosis, pre-existing stents in the femoral route, severe kinking or pre-existing aneurysms, and chronic dissections were also recorded. In case of any of these critical conditions, the patient was led to a transapical access route.

The following prostheses were used as valve systems: Edwards Sapien XT, Edwards Sapien 3 (Edwards Lifesciences, Irvine, CA, USA), Medtronic CoreValve, Medtronic EvolutR, and Medtronic EvolutPRO (Medtronic, Dublin, Ireland), as well as Symetis and Symetis accurate neo (Symetis SA, Ecublens, Switzerland). Due to the different valve systems, it was also recommended to use different sheath systems with a diameter of 14 French up to 22 French.

All implantations were performed in a hybrid OR by cardiologists and cardiac surgeons side by side after a final discussion in the “heart team”.

The peripheral vascular event rate was divided into major and minor complications according to the VARC criteria. Fatal (Typ 5), major (Typ 3a-c), and minor (Typ1-2) bleeding were recorded according to the BARC criteria. The primary, the combined endpoint, was defined as the occurrence of major complications according to the VARC criteria and/or fatal and major bleeding according to the BARC criteria. Predictors of the occurrence of this endpoint were considered separately for both groups in order to identify possible risk factors. Finally, the complication rate over time was observed for both groups in order to identify a possible learning effect of the procedure.

### 2.1. Surgical Approach

After verifying the indication for transfemoral TAVR by the Heart team, the access site was selected based on the anatomical conditions such as vessel diameter, tortuosity, and calcification detected by the CT-Scan. The inguinal vessel’s preparation took place after 3–4 cm long transversal skin incision two transverse fingers above the inguinal fold; optimal surgical visibility was achieved by proximal and distal vessel loops (Figure 1).

After that, optimal vascular palpation was possible. Additionally, heavy calcifications or vascular wall hematomas may occur after, e.g., previous angiographies that sometimes lead to a change on another place of the vessel, the contralateral side, or a transapical approach. If a sufficient artery was visible and palpable, the femoral artery was punctured far from the bifurcation in the middle of tiny purse-string sutures with 6-0 polypropylene. After insertion of the implantation sheath, the surgeon guarantees permanent control of the vascular situation to detect bleeding, sheath dislocation, or excessive pressure or tension in the femoral access area during the procedure. Before removing the implantation sheath at the end of the procedure, after angiographic control of the iliacal arteries to exclude relevant vascular injury, the femoral artery was closed by means of the prepared purse-string sutures with 6-0 polypropylene. A careful inspection of the vessel for possible intimal flap or plaque, which can contribute to stenosis after vascular occlusion, was performed. Finally, the distal pulse was palpated, and final angiographic control of the distal outflow was performed. The inguinal closure takes place with the facultative insertion of a Redon-drainage for 1–3 days.

### 2.2. Closure Device

The ProGlide closure device is a 6 Fr, two nitinol needle-guided suture-mediated closure system designed for closing the femoral artery access. For sheath sizes >8 Fr, a double ProGlide technique is recommended. Details on closure techniques and devices have been described previously [8].

### 2.3. Statistical Analysis

After dividing the collective into a surgical and ProGlide group, the Shapiro-Wilk test for normal distribution was carried out. All continuous variables were summarized as mean ± SD, the categoric variables were summarized as frequencies (%). Continuous variables were analyzed by t-test and categoric variables using the Mann-Whitney test. Binary variables were examined using the Chi-square test.

Predictors of complications were examined using logistic regression and documented along with OR, 95%-confidence intervals (CI), and *p*-value.

Missing variables were ignored. For the test of complication rates over the observation time, the data from the previous years were examined in the same way by using the Chi-square test.

For all of these calculations, we used XLSTAT (Addinsoft, New York, NY, USA) and IBM SPSS (IBM, Armonk, New York, NY, USA).

## 3. Results

Baseline and periprocedural characteristics are shown in Table 1 and Table 2 and give an overview of the typical TAVR patient collective. The Euro score I in both groups was significantly different, with a trend towards intermediate risk in the ProGlide group (Surgical group 21.5%, ProGlide group 18.9%, *p* = 0.01). More than 50% of the patients were women.

There was better kidney function, a higher pre-existing peripheral artery disease (PAD) rate, and a better LV function in the ProGlide group. Besides, the NYHA class of patients treated with the percutaneous closure system was significantly lower than in the surgical group. In contrast, the patients in the surgical group were significantly more often diabetic.

Due to the valve developments in the past few years, the systems used in both groups were different. As expected, the further reduction in the sheath diameter due to the further development of the sheath systems led to a significantly different diameter in our analysis (Surgical group 16.7 F vs. ProGlide group 15.1 F, *p* < 0.001). A very low conversion to surgery frequency can be detected (Surgical group 1.2%, ProGlide group 0.2%, *p* = 0.09).

The procedure time was significantly different in favor of the percutaneous collective (*p* < 0.001); however, the patients with surgical cut-down of the groin could be discharged earlier from the clinic (Surgical group 11.0 days vs. ProGlide group 12.6, *p* = 0.01). There is no significant difference in intra-hospital mortality (Surgical group 3.8% vs. ProGlide group 2.2%, *p* = 0.18), with only 23 patients dying during the hospital stay.

Overall, vascular complications occurred in the surgical group in 19.8%, while complications were observed in the ProGlide group in 23.8% (*p* = 0.18). The majority of these complications were limited to harmless minor bleeding according to the BARC criteria (Surgical group 8.9%, ProGlide group 16.9%, *p* = 0.001) or minor complications according to the VARC criteria (Surgical group 5.6%, ProGlide group 3.1%, *p* = 0.083).

The defined endpoint from major vascular complication and fatal or major bleeding according to VARC/BARC criteria was observed in 5.3% of the surgical group and 5.1% in the ProGlide group (*p* = 0.899) Table 3.

Possible predictors–those who reached a level of significance of complications cannot be identified in the surgical group. In the ProGlide group, there was a significantly increased incidence of complications with pre-existing PAD (OR 3.28, 95% CI 1.09–9.38 *p* = 0.03). Likewise, women were increasingly affected by a complication (OR 10.17, 95% CI 2.70–38.28 *p* = 0.001) Table 4.

**Figure 2:**
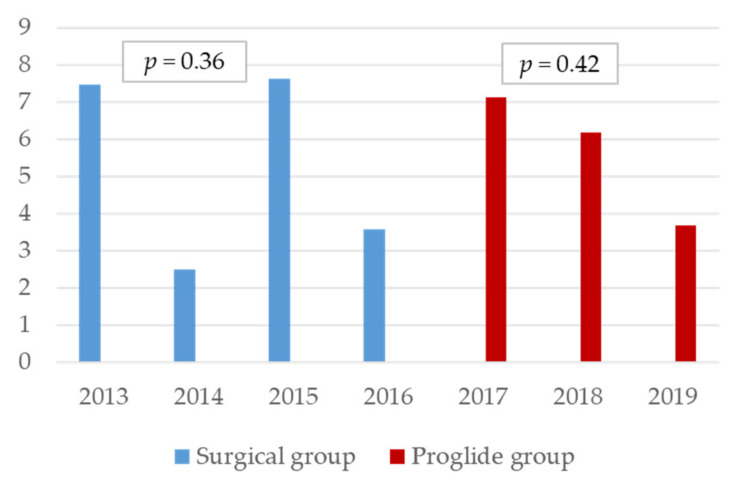
Occurence of the primary endpoint over time (expressed as percentage per year).

The sheath size nor the patient’s BMI, like the age, does not appear to be a risk of complications in the ProGlide or surgical groups. Finally, in the presence of the primary endpoint, the hospital stay in both groups was significantly increased (Surgical group OR 1.04, 95% CI 1.01–1.08 *p* = 0.01; ProGlide group OR 1.06, 95% CI 1.02–1.10 *p* = 0.001), while the duration of the procedure is only significantly extended in the ProGlide group (OR 1.02, 95% CI 1.01–1.103 *p* = 0.01). Looking at the complication rates over time, interestingly, no significant decrease in the ProGlide or surgical groups could be observed (Figure 2).

## 4. Discussion

The vascular access route, which was routinely performed by surgical cut-down at the beginning of the TAVI era in our institution, changed continuously as various percutaneous closure systems increased. Studies that provide a direct comparison between percutaneous and surgical procedures are rare.

Against the background of providing low-risk patients, it is increasingly important to focus on aspects such as vascular access security. Spitzer et al. showed better access control and lower bleeding rates in the surgical collective in 334 patients than in the percutaneous group [9]. Kawashima et al. were able to treat primarily 586 patients who also responded to propensity matching in similar collectives that were grouped—166 patients in each group—showing that the percutaneous collective had a significant advantage in terms of bleeding rate and major complications [10]. Smaller case series as described by Holper et al. could initially indicate that the percutaneous procedure was not inferior to the surgical procedure [11], while larger studies confirmed this suggestion [12,13]. The rate of absolute major complication rates for the surgical procedure was given in these studies up to 20%. Drafts et al. described, in the same setting, that the ProGlide approach was associated with a shorter hospital length of stay and a similar risk of vascular complications and all-cause mortality at 12 months [14]. The benefit of our study is that between 2013 and 2016, the treatment was exclusively surgical. In 2017, there was a switch to the percutaneous approach. Therefore, there is no possible bias in patient selection. On the other hand, we only compared one percutaneous device with the surgical procedure. Here, the comparison was made specifically with the Perclose ProGlide™ system, which prevailed in a head-to-head comparison with the also used ProStar™ device (Abbott, Chicago, IL, USA) [15,16].

In our surgical group, a major vascular complication rate of <5% could be realized, although we refer to the limitation of a single-center study, and it is a retrospective survey of our current TAVR program. Nevertheless, our “Nuremberg surgical Approach” seems to have a significantly lower complication rate, which we most likely attribute to the listed surgical technique [17].

The potential predictors of major complications shown in our ProGlide group analysis also made it clear that some patients may benefit suggestively from a surgical approach. Our analysis showed that women and patients with pre-existing PAD could significantly experience a major vascular complication. In percutaneous way at the time of puncture, the vessel is always more blind than the surgical one. Roadmap images, ultrasound-guiding, and preprocedural reconstruction of the vessel from the CT images may yield potential enough to close this gap in the future. Additionally in our Center, we believe that these will be the method of choice to grant a proper visualization of the femoral bifurcation, calcium distribution and in case of a closure-device, use central needle entry as described in other studies [18].

Limitations of the study: the retrospective study design is the first limitation. Secondly, the data is based on a single Center cohort. However, the difference between groups in terms of prevalence in peripheral artery disease is another limitation. On the other hand, this difference highlights the good results obtained with the surgical approach in this high-risk patient category.

Conclusion: we can conclude that, on the statistical side, we do compare apples and oranges, but on the “safety” side, we clearly showed that the surgical approach could transform a high-risk group due to a peripheral artery disease to a lower risk with the same incidence of vascular complications as the no-PAD patients.

Further studies are needed to identify patients who benefit especially from one of the possible access procedures to make the TAVR access an individual one. It is gratifying to see that no different in-hospital mortality can be seen in our two groups after reaching a relevant vascular complication. However, data from long-term outcomes after such an event are well known with increased mortality over time. This again shows that more data is needed to protect patients from these complications. On the other hand, the prolonged hospital stay after a complication is important not only from an economic point of view. A longer in-hospital stay was associated with a higher rate of complications such as thromboembolic complications and infections in our geriatric patients.

Our data shows that it will continue to be necessary, in addition to the currently established percutaneous procedure, to have the surgical cut down available in each TAVR center and to use it individually in order to improve the outcome of TAVR patients further.

## Figures and Tables

**Table 1:** Baseline characteristics.

Variable	Surgical Group (*n* = 338)	Proglide Group (*n* = 449)	*p*-Value
Age (y)	81.4 ± 6.1	81.0 ± 5.6	0.718
female Sex	188 (55.6%)	229 (51.0%)	0.913
BMI	27.1 ± 4.6	27.3 ± 5.1	0.712
GFR	45.7 ± 18.6	57.7 ± 22.7	<0.001
PAD	63 (18.6%)	121 (26.9%)	0.006
Redo-procedure	64 (18.9%)	68 (15.1%)	0.159
after CABG	46 (13.6%)	50 (11.1%)	0.294
after AVR	15 (4.5%)	18 (4.0%)	0.766
COPD	60 (17.7%)	83 (18.5%)	0.792
DM	122 (36.1%)	127 (28.3%)	0.002
NYHA	3.0 ± 0.5	2.7 ± 0.9	<0.001
LV-EF	3.3 ± 1.0	3.4 ± 1.0	0.018
AR	1.2 ± 0.7	1.0 ± 0.8	<0.001
MR	1.4 ± 0.6	1.3 ± 0.6	0.148
log. ES I	21.5 ± 14.8	18.9 ± 12.6	0.01

Data are expressed as mean ± standard deviation or total number with percent (%). BMI, body mass index atrial; GFR, glomerular filtration rate; PAD, peripheral artery disease; CCS, Redo-procedure, total of all previous cardiac surgery operations; CABG, coronary artery bypass grafting; AVR, aortic valve replacement; COPD, chronic obstructive pulmonary disease; DM, diabetes mellitus; NYHA, New York Heart Association, stages I–IV are indicated numerically; LV-EF, left ventricular ejection fraction; AR, aortic regurgitation; MR, mitral regurgitation; log. ES I, logistic EuroScore I.

**Table 2:** Procedural characteristics.

Variable	Surgical Group (*n* = 338)	Proglide Group (*n* = 449)	*p*-Value
Edwards SapienXT	86 (25.4%)	2 (0.4%)	<0.001
Edwards Sapien 3	156 (46.2%)	268 (59.7%)	<0.001
Boston Symetis acurate	43 (12.7%)	136 (30.3%)	<0.001
Medtronic CoreValve	33 (9.8%)	0 (0%)	-
Medtronic EvolutR	20 (5.9%)	21 (4.7%)	0.438
Medtronic EvolutPRO	0 (0%)	22 (4.9%)	-
valve-in-valve procedure	15 (4.4%)	27 (6.0%)	0.330
Sheath size (Fr)	16.7 ± 2.8	15.1 ± 1.6	<0.001
Sheath ≥ 18 Fr	163 (48.2%)	74 (16.5%)	<0.001
Valve size (mm)	25.7 ± 2.5	25.4 ± 2.4	0.101
Predilatation	319 (94.4%)	423 (94.2%)	0.919
Postdilatation	118 (34.9%)	107 (23.8%)	0.001
conversion to surgery	4 (1.2%)	1 (0.2%)	0.093

Data are expressed as mean ± standard deviation or total number with percent (%). Valve-in-valve procedure, each TAVI implantation in a previously surgically implanted aortic valve; Sheath size, TAVI implantation Sheath, size in French (Fr); Valve size, Size of the TAVI valve in millimeter (mm); Predilatation, balloon valvuloplasty before TAVI implantation; Postdilatation, post dilatation of the TAVI valve; conversion to surgery, any event that leads to a thoracotomy as part of the procedure.

**Table 3:** Postoperative Characteristics.

Variable	Surgical Group (*n* = 338)	Proglide Group (*n* = 449)	*p*-Value
all complications	67 (19.8%)	107 (23.8%)	0.180
primary endpoint	18 (5.3%)	23 (5.1%)	0.899
secondary endpoint	29 (8.6%)	33 (7.3%)	0.526
VARC-2			
major vascular complication	13 (3.8%)	11 (2.4%)	0.259
minor vascular complication	19 (5.6%)	14 (3.1%)	0.083
BARC			
fatal bleeding	1 (0.3%)	1 (0.2%)	0.840
major bleeding	4 (1.2%)	11 (2.4%)	0.198
minor bleeding	30 (8.9%)	76 (16.9%)	0.001
procedure time (min)	89.1 ± 40.3	68.2 ± 22.9	<0.001
hospital stay (d)	11.0 ± 8.8	12.6 ± 8.1	0.013
hospital mortality	13 (3.8%)	10 (2.2%)	0.182

Data are expressed as mean ± standard deviation or total number with percent (%). All complications, sum of all peripheral vascular complications; primary endpoint, sum of fatal bleeding, major bleeding and major vascular complications, secondary endpoint, primary endpoint plus hospital mortality; VARC-2, valve academic research consortium, 2 consensus document; BARC, bleeding academic research consortium.

**Table 4:** Predictors of occurrence of the combined primary endpoint in the surgical and ProGlide groups.

		Surgical Group			Proglide Group	
Predictor	OR	95%CI	*p*	OR	95%CI	*p*
Age	0.94	0.87–1.01	0.10	1.03	0.93–1.13	0.27
female sex	0.82	0.28–2.39	0.72	0.72	2.70–38.28	0.001
BMI	0.98	0.87–1.11	0.77	0.92	0.83–1.02	0.13
GFR	1.00	0.97–1.02	0.71	1.01	0.98–1.04	0.57
NYHA	1.47	0.53–4.07	0.46	1.58	0.83–2.98	0.16
LV-EF	1.05	0.59–1.88	0.87	0.73	0.43–1.22	0.23
log. ES I	0.98	0.93–1.04	0.55	0.95	0.89–1.01	0.12
PAD	0.85	0.19–3.77	0.83	3.28	1.09–9.83	0.03
Redo procedure	2.42	0.56–10.40	0.23	1.71	0.33–8.78	0.52
valve-in-valve procedure	2.38	0.08–71.71	0.62	3.09	0.42–22.49	0.27
COPD	1.36	0.37–4.92	0.64	1.32	0.38–4.62	0.66
DM	1.82	0.62–5.34	0.27	2.00	0.76–5.29	0.16
Sheath size	0.94	0.50–1.77	0.42	0.97	0.51–1.84	0.93
Sheath > 18 Fr	1.12	0.03–42.49	0.95	1.17	0.10–13.67	0.90
predilatation	2.77	0.13–60.61	0.52	7.64	0.29–204.59	0.23
postdilatation	0.52	0.19–1.46	0.22	1.46	0.59–3.62	0.41
procedure time	1.00	0.99–1.01	0.48	1.02	1.01–1.03	0.01
hospital stay	1.04	1.01–1.08	0.01	1.06	1.02–1.10	0.001
hospital mortality	2.22	0.41–11.93	0.35	0.59	0.03–11.41	0.73

Separate analysis of each group with respect to the combined endpoint. Data are expressed as Odds Ratio (OR) with 95% confidence interval a *p*-value. BMI, body mass index atrial; GFR, glomerular filtration rate; NYHA, New York Heart Association; LV-EF, left ventricular ejection fraction; log. ES I, logistic EuroScore I; PAD, peripheral artery disease; Redo-procedure, total of all previous cardiac surgery operations; COPD, chronic obstructive pulmonary disease; DM, diabetes mellitus.

## Data Availability

The data presented in this study are available on request from the first author. The data are not publicly available due to data protection regulations.
